# Active Pore-Edge Engineering of Single-Layer Niobium Diselenide Porous Nanosheets Electrode for Hydrogen Evolution

**DOI:** 10.3390/nano9050751

**Published:** 2019-05-16

**Authors:** Jianxing Wang, Xinyue Liu, Ying Liu, Guowei Yang

**Affiliations:** State Key Laboratory of Optoelectronic Materials and Technologies, Nanotechnology Research Center, School of Materials Science & Engineering, School of Physics, Sun Yat-sen University, Guangzhou 510275, Guangdong, China; wangjx46@mail2.sysu.edu.cn (J.W.); liuxiny8@mail2.sysu.edu.cn (X.L.); liuying35@mail.sysu.edu.cn (Y.L.)

**Keywords:** TMDs, niobium diselenide, hydrogen evolution, electrocatalysis

## Abstract

Two-dimensional transition-metal dichalcogenides (TMDs) possess interesting catalytic properties for the electrochemical-assisted hydrogen-evolution reaction (HER). We used niobium diselenide (NbSe_2_) as a representative TMD, and prepared single-layer NbSe_2_ porous nanosheets (PNS) by a double-sonication liquid-phase exfoliation, with H_2_O_2_ as a pore-forming agent. The single-layer NbSe_2_ PNS were drop-cast on carbon foam (CF) to fabricate a three-dimensional robust NbSe_2_ PNS/CF electrode. The NbSe_2_ PNS/CF electrode exhibits a high current density of −50 mA cm^−2^ with an overpotential of 148 mV and a Tafel slope of 75.8 eV dec^−1^ for the HER process. Little deactivation is detected in continuous CV testing up to 20,000 cycles, which suggests that this novel NbSe_2_ PNS/CF is a promising catalytic electrode in the HER application. The porous structure of single-layer NbSe_2_ nanosheets can enhance the electrochemical performance compared with that of pore-free NbSe_2_ nanosheets. These findings illustrate that the single-layer NbSe_2_ PNS is a potential electrocatalytic material for HER. More importantly, the electrochemical performance of the NbSe_2_ PNS/CF expands the use of two-dimensional TMDs in electrocatalysis-related fields.

## 1. Introduction

The energy crisis has aroused extensive research interest in the search for sustainable energy-conversion systems that exhibit a high productivity and low cost. Hydrogen (H_2_) is one of the most promising candidates to replace fossil fuels in the future [1,2,3,4,5,6,7,8]. The electrochemical hydrogen-evolution reaction (HER) is considered to be the most important and promising route to produce hydrogen [9,10,11,12,13]. Platinum (Pt) and its alloys are the most electrochemically active and stable catalysts for HER. However, the high price and limited availability of Pt prevent its large-scale usage in practice [14]. Therefore, the development of nonprecious-metal catalysts that drive HER at a low overpotential with an excellent reaction efficiency is essential for large-scale production of hydrogen through electrochemical water splitting [15,16].

Recently, two-dimensional (2D) transition-metal dichalcogenides (TMDs), such as MoS_2_ and WS_2_, have attracted much attention because of their layer structure and excellent electrocatalytic properties [17]. The inherent contact resistance of TMD materials has not yet been optimized, especially for the trigonal prismatic (2H) basal plane. The crystalline strain and metallic octahedral (1T) sites are both important factors to modulate the catalytic activity of TMD nanosheets [18,19,20]. Therefore, an improvement of the conductivity and creation of active edge sites of the TMDs are expected to achieve a better HER performance.

In the thermodynamically stable 2H phase, MoS_2_, MoSe_2_, WS_2_ and WSe_2_ are semiconductors [21]. The NbSe_2_ belongs to the Group V transition metal dichalcogenides. NbSe_2_ has a similar crystalline structure to MoS_2_ and WS_2_. However, the NbSe_2_ TMDs with metallic conductivity have stolen the limelight [22]. The electrical resistivity of NbSe_2_ is only 10^−4^ Ω·cm, which is six orders of magnitude less than that of MoS_2_ [23]. The Group V NbSe_2_ TMDs are prized for their low-dimensional crystal structure and exhibit interesting electronic properties, such as superconductivity, charge density waves and Mott transition [24]. The layers of NbSe_2_ are stacked together through Van der Waals interactions and can be exfoliated into thin layers. First-principles calculations have suggested that single-layer NbSe_2_ has a charge density wave phase with a different periodicity compared with that of the bulk, as well as a larger gain of electronic energy, which result in a higher transition temperature [25]. However, the electrochemical and electrocatalytic properties of the single-layer Group V TMDs have not been well established.

Here, we prepared 3D single-layer NbSe_2_ porous nanosheets as advanced HER electrocatalysts. Strategies have been developed to promote the HER catalytic effect of NbSe_2_, which can increase the number of edge active sites significantly [4,26,27] and improve the electrical conductivity [28]. We fabricated single-layer NbSe_2_ porous nanosheets/carbon-foam electrode, which exhibited a Tafel slope of 75.8 eV dec^−1^ and an overpotential of −148 mV at a current density of −50 mA cm^−2^ in the HER process. The as-revealed catalytic performance of a single-layer NbSe_2_ PNS/carbon foam (CF) electrode outperforms most of the previously reported non-noble HER catalysts, such as MoS_2_-NbSe_2_ hybrid nanobelts with a Tafel slope of 79.5 eV dec^−1^ and an overpotential of −410 mV at a current density of −10 mA cm^−2^ [20], three-dimensional molybdenum sulfide sponges with a Tafel slope of 185 eV dec^−1^ and an overpotential of −30 mV at a current density of −10 mA cm^−2^ [29], and a three-dimensional MoS_2_/GO framework with an overpotential of −210 mV at a current density of −10 mA cm^−2^ [30]. Little deactivation has been detected in stability testing, even up to 20,000 cycles, which reveals the promising prospect of this novel single-layer NbSe_2_ porous nanosheets/carbon in massive electrochemical water splitting and hydrogen production.

## 2. Materials and Methods

NbSe_2_ pristine powder (99%, Alfa Aesar, Shanghai, China), sodium cholate (NaC) (99%, Alfa Aesar, Shanghai, China), Nb_2_O_5_ powder (99.99%, Alfa Aesar, Shanghai, China), carbon foam, Nafion solution (5 wt.%, Alfa Aesar, Shanghai, China), and state-of-the-art Pt-C (10 wt.% Pt, Alfa Aesar, Shanghai, China). Other chemicals were from SinoPharm (Shanghai, China) and used without further purification.

NbSe_2_ powder (starting concentration C_i_ = 8 mg mL^−1^) was dissolved in 200 mL of aqueous NaC solution (C_NaC_ = 4 mg mL^−1^). To obtain a stable single-layer nanosheets dispersion and to avoid re-stacking, the initial mass ratio of NaC to TMD (C_NaC_/C_i_) was kept at ~0.5, which is the optimized surfactant concentration ratio to drive efficient exfoliation. The initial dispersion was sonicated for 6 h at a 30% amplitude under pulsed mode with 2 s on and 2 s off while chilled using a double-jacketed water-cooling system and chiller. The resultant raw dispersions were subjected to a brief centrifugation step (TGL-16 centrifuge, Xiangyi Co. Ltd., Hunan, China) at 5000 rpm for 50 min to remove un-exfoliated material. The upper suspension was then subjected to another centrifugation step at 10,000 rpm for 30 min to separate the single-layer nanosheets (NSs) from the few-layer NSs. The collected single-layer nanosheets were sonicated again using the same conditions, except in a 2.5 vol% H_2_O_2_ to create pores on the nanosheets. Finally, the NbSe_2_ single-layer PNS were rinsed with 1200 mL of water to remove residual surfactant and H_2_O_2_ during the vacuum filtration.

Scanning electron microscopy (SEM, Auriga-4525, Carl Zeiss Inc., Oberkochen, Germany) and transmission electron microscopy (TEM, Tecnai G2 F30, FEI Inc., Eindhoven, The Netherlands, operated at 300 kV) were used to identify the morphology of the as-synthesized samples. To understand the surface chemical states of the superficial bonded elements, X-ray photoelectron spectroscopy with a monochromatic Al-Kα source (XPS, ESCA Lab250. Thermo Scientific, East Grinstead, UK) was conducted. Ultraviolet photoelectron spectroscopy (UPS, VG ESCALAB Mk II, Thermo Scientific, East Grinstead, UK) was performed using He I (21.2 eV) resonance line. The X-ray diffraction (XRD) pattern was recorded on a Rigaku D-MAX 2200 VPC (Rigaku Co., Tokyo, Japan) diffractometer with Cu-Kα as the radiation source (λ = 0.154 nm). Atomic force microscopy (AFM) images were obtained by using a Bruker Multimode V8 system (Dimension icon, Bruker Inc., Billerica, MA, USA) with the tapping mode after the samples had been deposited on a freshly cleaved mica surface by spin coating.

Typically, 5 mg of sample and 30 μL of Nafion solution (5 wt.%) were dispersed uniformly in 1 mL of a water–ethanol solution with a volume ratio of 4:1 by sonicating for 0.5 h to form a homogeneous ink. Then, 100 μL catalyst ink was loaded onto a carbon-foam electrode with a geometric area of 0.5 cm^−2^. The catalytic performances of the single-layer NbSe_2_ PNS/CF for HER were studied using a three-electrode configuration connected to a CH Instrument workstation at room temperature (25 °C). The NbSe_2_ PNS/CF electrode was used as the working electrode. An Ag/AgCl (sat. KCl) electrode and a graphite rod were used as the reference and counter electrodes, respectively. All measurements were performed in 0.5 M H_2_SO_4_ (aq.). All reported potentials were referenced to the reversible hydrogen electrode (RHE) through RHE calibration according to: E (RHE) = E^θ^(Ag/AgCl sat.) + 0.198 + 0.059 pH. The polarization curves were obtained by sweeping the potential from −0.4 to 0.2 V versus the RHE at room temperature with a sweep rate of 5 mV s ^−1^. The electrochemical impedance spectroscopy (EIS) measurements were performed in the same configuration at an open circuit potential of 210 mV over a frequency range from 100 kHz to 0.1 Hz at an amplitude of 2 mV. The resistance of 0.5 M H_2_SO_4_ is ∼15 Ω, which was determined by EIS.

## 3. Results

**Figure 1:**
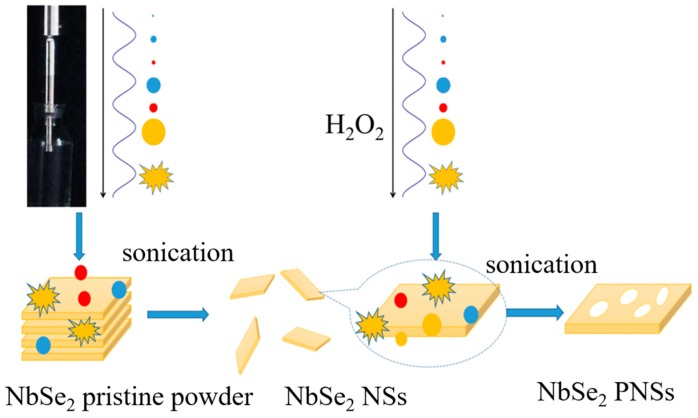
Schematic illustration of the process to prepare the single-layer porous NbSe_2_ nanosheets.

Liquid-phase sonication exfoliation is a powerful and scalable technique to produce few-layer TMD nanosheets [31,32,33,34,35]. Figure 1 illustrates the fabrication process of the single-layer NbSe_2_ PNS. Firstly, the NbSe_2_ pristine powders were exfoliated into few-layer nanosheets through the sonication liquid-phase exfoliation process. Secondly, porous structures in the plane of the prepared nanosheets were constructed through a second liquid-phase sonication process in H_2_O_2_. After double liquid-phase sonication, NbSe_2_ crystals in the powder were exfoliated into single-layer PNS.

**Figure 2:**
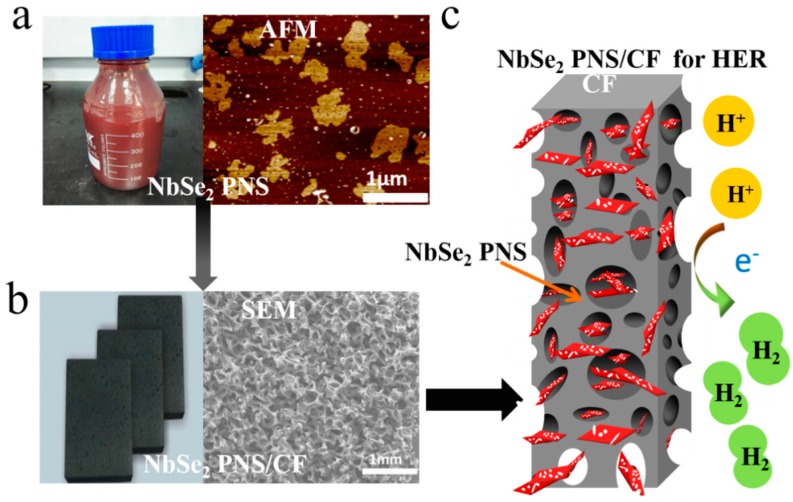
(**a**) Photograph of well dispersed NbSe_2_ porous nanosheets (PNS) solution and AFM image of scattered NbSe_2_ PNS. (**b**) Optical photograph and corresponding SEM image of as-developed NbSe_2_ PNS/carbon foam (CF) electrode. (**c**) Illustration of the NbSe_2_ PNS/CF electrode toward the hydrogen-evolution reaction (HER).

NbSe_2_ powders tend to have a bulk structure with fewer edge sites, but single-layer NbSe_2_ PNS are almost 1 nm thickness and contain many holes in the plane. Therefore, the edge active sites of single-layer NbSe_2_ PNS are several orders of magnitude higher than those of the NbSe_2_ powders. The NbSe_2_ PNS solution which mixed with Nafion was drop-casted on the carbon foam (CF) to fabricate the NbSe_2_ PNS/CF electrode (Figure 2). The macropore-like structure of CF could increase the contact area of catalyst and electrolyte as well as improve the electrochemical property of the NbSe_2_ PNS [36].

**Figure 3:**
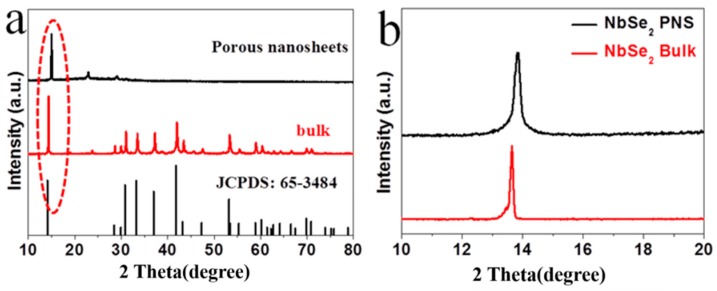
XRD patterns of (**a**) NbSe_2_ PNS and pristine powder NbSe_2_; (**b**) the amplifying district of circle in (**a**).

To confirm this hypothesis, single-layer and porous nanosheets were investigated via X-ray powder diffraction (XRD) to identify the corresponding crystal structure. According to Figure 3a, several peaks of bulk NbSe_2_ are assigned to the hexagonal 2H-NbSe_2_ (JCPDS 65-3484). As a comparison, the NbSe_2_ PNS exhibit an obvious diffraction peak at 14.1°, which is related to the (002) peak of hexagonal NbSe_2_. In addition, the (002) peak of NbSe_2_ PNS shifts to the higher angle compared to that of bulk NbSe_2_ due to the partial transformation from 2H-NbSe_2_ to 1T-NbSe_2_ (Figure 3b). The similar phenomenon of 2H and 1T MoS_2_ monolayer has been reported in literature [37]. Besides this, the positive shift peak of NbSe_2_ PNS suggests the highly exfoliated effect of the NbSe_2_ nanosheets and the widened interlayer spacing of NbSe_2_ PNS owing to the strong exfoliating ability with the assistance of H_2_O_2_.

**Figure 4:**
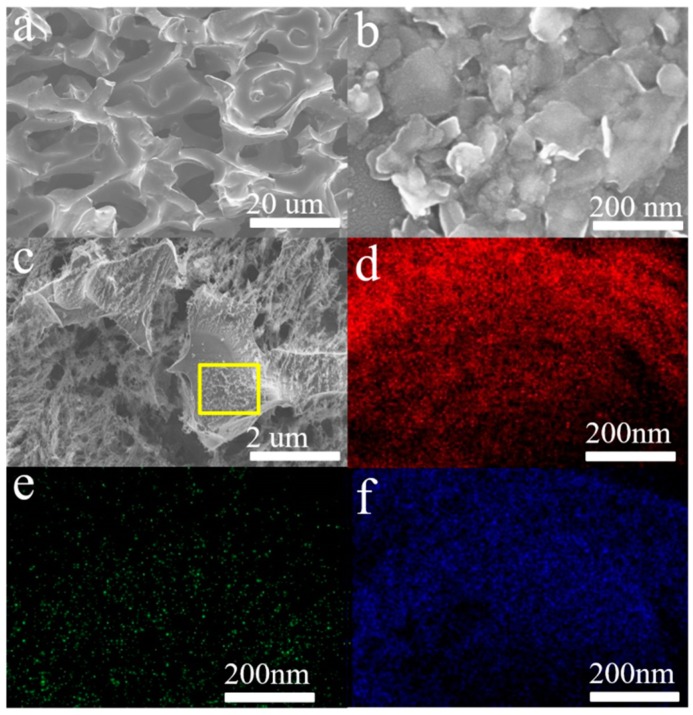
SEM images of the (**a**) bare carbon foam, (**b**) as-synthesized NbSe_2_ PNS and (**c**) 3D NbSe_2_ PNS/CF. (**d**–**f**) EDS mapping of Nb, Se, and C elements on the surface of NbSe_2_ PNS/CF (the region marked in (**c**)).

Figure 4a and Figure S1a reveal that the CF support exhibits a network porous structure, and the size of the holes ranges from dozens to hundreds of micrometers. The initial NbSe_2_ pristine powder is in the form of crystalline flakes with size of a few to dozens of micrometers (Figure S1b). Furthermore, the size of the NbSe_2_ PNS are smaller than that of the NbSe_2_ NS (Figure S1c,d), suggesting that the second liquid-phase sonication further broke the NbSe_2_ nanosheets to smaller pieces. The elemental distributions indicate the uniform distribution of elemental C, Nb and Se on the 3D NbSe_2_ PNS/CF surface, meaning that the NbSe_2_ PNS have been attached onto the CF (Figure 4d–f).

**Figure 5:**
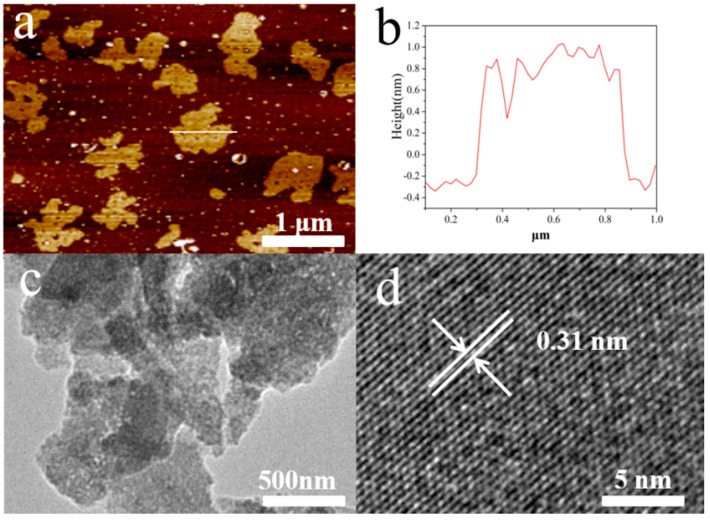
(**a**) AFM image and (**b**) the corresponding thickness distribution of NbSe_2_ PNS. (**c**) TEM and (**d**) HR-TEM images of NbSe_2_ PNS.

The thickness of the NbSe_2_ PNS was investigated by atomic force microscopy (AFM) and TEM. The AFM results in Figure 5a,b confirmed that single-layer NbSe_2_ PNS with thickness of ~1 nm were obtained. In comparison, the NbSe_2_ NSs thickness (~1.5 nm) is larger than the NbSe_2_ PNS (Figure S2d). The porous structure can also be clearly seen from Figure 5c, due to the etching effect of H_2_O_2_. The size of the nanosheets’ hole ranges from several to dozens of nanometers. The high-resolution TEM (HRTEM) image (Figure 4d) shows the lattice fringe of 0.31 nm was resulting from the (002) crystal planes of NbSe_2_. However, as shown in Figure S2, NbSe_2_ NSs without etching by H_2_O_2_ do not exhibit a hole structure in the nanosheets. Relative to the NbSe_2_ powder, the unsaturated edges of the exfoliated porous NbSe_2_ PNS structure are more active for proton adsorption and thus enhance the HER performance [28].

**Figure 6:**
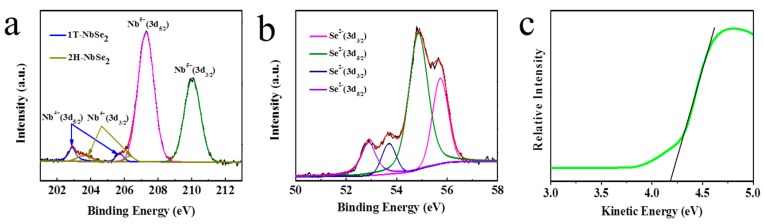
The high-resolution XPS spectra of single-layer NbSe_2_ PNS. (**a**) Se 3d and (**b**) Nb 3d spectra. (**c**) UPS spectrum of single-layer NbSe_2_ PNS.

XPS was used to characterize the chemical composition and binding energy of the single-layer NbSe_2_ PNS and NbSe_2_ NSs. The XPS spectrum of Nb 3d for the single-layer NbSe_2_ PNS is shown in Figure 6a. The high-resolution spectrum shows 1T-NbSe_2_ peaks (blue line) around 203.2 and 206.0 eV, which corresponds to the Nb^4+^ 3d component. 2H-NbSe_2_ peaks (dark yellow line) at around 204.1 and 206.5 eV correspond to the Nb^4+^ 3d component. Peaks around 207.8 and 210.1 eV are attributed to Nb^5+^, indicating that oxidized valence of Nb^5+^ exists at the surface of nanosheets. The high-resolution XPS spectra of O 1s of NbSe_2_ PNS (Figure S7) shows two peaks at 529.6 and 532.0 eV, which are due to the lattice oxygen and the adsorption oxygen in the surface of catalyst, respectively. The high-resolution XPS spectrum of Se 3d for the single-layer NbSe_2_ PNS is shown in Figure 6b. Peaks at around 52.85 eV and 53.65 eV are attributed to Se 3d_5/2_ and Se 3d_3/2_ of 1T-NbSe_2_, respectively. Another two peaks at 54.9 eV and 55.7 eV can be assigned to Se 3d_5/2_ and Se 3d_3/2_ of 2H-NbSe_2_ [38]. In comparison, according to Se 3d core-level peaks of the NbSe_2_ NSs in Figure S3a, 2H-NbSe_2_ peaks around 54.7 eV and 55.7 eV should be related to Se 3d_3/2_ and Se 3d_5/2_. The Nb 3d core-level peaks of the NbSe_2_ NSs at 203.5 eV and 206.6 eV in Figure S3b should represent the Nb^4+^ 3d component. However, the XPS of NbSe_2_ NSs is not detected in the 1T phase in the nanosheets, which shows that part of the 2H phase can be transformed to the 1T phase during the second sonication process with the assistance of H_2_O_2_ [38]. The UPS of single-layer NbSe_2_ PNS was shown in Figure 6c. The working function (Φ) of NbSe_2_ PNS was calculated as 4.18 eV. When kinetic energy is used as the *x*-axis, the equation of the working function is Φ = hγ − (E_Fermi,k_ − E_SE Cutoff, k_). The photon energy of XPS monochromatic is 1.486 eV and E_Fermi,k_ is 1.486 eV. Hence, the value of Φ is equal to the value of E_SE Cutoff, k_.

The XPS spectra of NbSe_2_ PNS after 50 consecutive cyclic voltammetry sweeps and NbSe_2_ PNS after 25 h stability test were shown in Figure S8. The related XPS analysis results of NbSe_2_ PNS, NbSe_2_ PNS after 50 consecutive cyclic voltammetry sweeps, as well as NbSe_2_ PNS after 25 h stability test were summarized in Table 1. The Se/Nb ratio gradually decreases, and the content of O increases during the long-term electrochemical test, which illustrates that the NbSe_2_ is oxidized into niobium oxide during the electrochemical test. The NbSe_2_ NSs exhibits Nb^4+^ in NbSe_2_ (Figure S3), and no oxygen was detected. Also, the introduction of H_2_O_2_ brought substantial oxygen group on the surface of NbSe_2_, causing a high O/Nb ratio of 3.2 in the NbSe_2_ PNS. The O/Nb ratio was further increased to 5.5 after electrochemical test due to the oxidization process of NbSe_2_ to Nb_2_O_5_ at acidic media with constant applied potential.

**Figure 7:**
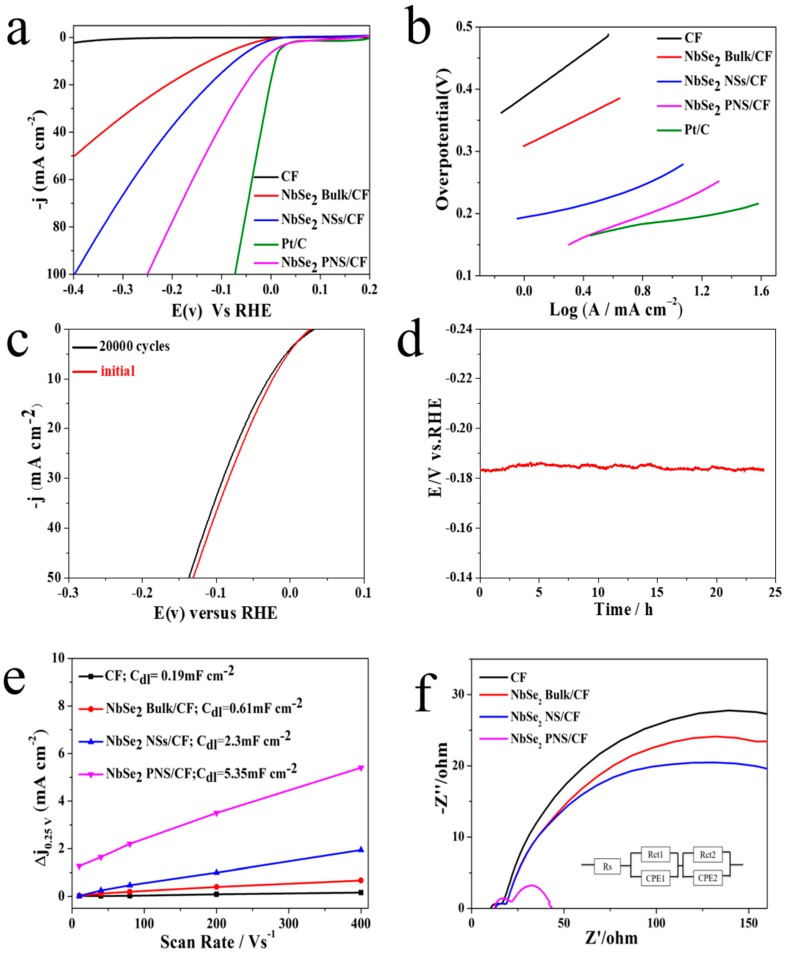
(**a**) LSV curves of the NbSe_2_ PNS/CF, NbSe_2_ NSs/CF, NbSe_2_ bulk/CF, Pt-C and bare CF at a scan rate of 5 mV s^−1^. (**b**) Tafel plots of the NbSe_2_ PNS/CF, NbSe_2_ NSs/CF, NbSe_2_ bulk/CF, Pt-C and bare CF. (**c**) Polarization curve comparison between initial and after 20,000 cycles of the NbSe_2_ PNS/CF at a scan rate of 50 mV s^−1^. (**d**) Chronopotentiometric curve recorded for the NbSe_2_ PNS/CF at a constant cathodic current density of 50 mA cm^−2^. (**e**) The slope of current density at open circuit potential (OCP) vs. scan rate. (**f**) Nyquist plots of NbSe_2_ PNS/CF, NbSe_2_ NSs/CF, NbSe_2_ bulk/CF and CF.

The electrocatalytic HER activities of the NbSe_2_ PNS/CF were investigated by linear-sweep voltammetry (LSV) using a standard three-electrode setup in 0.5 M H_2_SO_4_ solution with a scan rate of 5 mV s^−1^. For comparison, the reference commercial Pt-C (10 wt. % Pt) was studied under the same condition. Figure 7a shows the LSV curves of various samples after IR compensation. Pure CF shows limited HER activity within the potential range of −0.4~0.2 V (versus RHE), whereas the Pt-C has the best catalytic performance. To achieve current densities of −50 mA cm^−2^, the exfoliated porous NbSe_2_ PNS/CF requires an overpotential of 148 mV. In contrast, the NbSe_2_ NSs/CF without porous structure and the NbSe_2_ bulk exhibits an inferior HER activity with a larger overpotential of 242 mV and 400 mV to drive the hydrogen-evolution current of −50 mA cm^−2^, respectively. Compared with the HER activities of the NbSe_2_ PNS/CF, NbSe_2_ NSs/CF and NbSe_2_ bulk/CF, it can be concluded that the porous nanosheets structure of the single-layer NbSe_2_ PNS indeed improve the catalyzed HER activity. A similar phenomenon has been reported in ultra-thin and porous MoSe_2_ nanosheets [39].

To understand the high HER activity of the NbSe_2_ PNS/CF, Tafel plots of various electrodes were studied (Figure 7b). The Tafel plots were derived from the quasi-static polarization curve to reflect the inherent mechanism of the HER process and the rate-determining step for the entire HER process. A smaller Tafel slope is referred to as a faster increase of hydrogen-generation rate [40]. The pure CF shows a large Tafel slope of ~300 mV dec^−1^ in the η range of 360–480 mV, which indicates that it is a less active HER catalyst. The Pt-C is the most active material with the smallest Tafel slope of 41 mV dec^−1^. The NbSe_2_ PNS/CF possesses a Tafel slope of 75.8 mV dec^−1^, which is smaller than those of 97.3 and 155 mV dec^−1^ for the NbSe_2_ NS/CF and NbSe_2_ bulk/CF, respectively, which demonstrates the more rapid HER kinetics of NbSe_2_ PNS/CF. The Tafel slope of NbSe_2_ PNS/CF is either close to or even better than the records of the three-dimensional TMD-based electrocatalysts (Table S1), such as MoS_2_-NbSe_2_ hybrid nanobelts (101.2 mV dec^−1^) [20], three-dimensional molybdenum sulfide sponges (185 mV dec^−1^) [29] and three-dimensional MoS_2_/GO frameworks (86.3 mV dec^−1^) [30]. The porous structure of the single-layer NbSe_2_ PNS can improve the catalytic activity toward better HER due to the additional edge sites along the margins of the hole. Furthermore, the unsaturated Se along the holes provides possible active sites for hydrogen-ion adsorption [28].

The excellent stability of electrocatalysts towards the HER is vital for future water-splitting systems. Figure 7c shows the continuous cycling performance of the NbSe_2_ PNS/CF electrode for 20,000 cycles at a scan rate of 50 mV s^−1^. At a current density of −50 mA cm^−2^, the overpotential of the NbSe_2_ PNS/CF shows a slight increase after 20,000 cycles. Consequently, the NbSe_2_ PNS/CF exhibits an ultra-high activity and a satisfied long-term cycle stability. Figure 7d shows the chronopotentiometric plot recorded for the NbSe_2_ PNS/CF at a constant current density of −50 mA cm^−2^. The potential of NbSe_2_ PNS/CF was maintained constant with little oscillation over 24 h, suggesting the high durability of the NbSe_2_ PNS/CF. The SEM and TEM images (Figure S5) show that the NbSe_2_ PNS maintain a 2D lamella structure and regular lattice fringes of 0.31 nm. The NbSe_2_ PNS after 20,000 cycles were surface partly oxidized to niobium pentoxide (Figure S4). The diffraction peaks were labelled as well numbers were assigned to the crystal planes of Nb_2_O_5_ (Figure S9, JCPDF 72-1121) while the diffraction peaks of NbSe_2_ PNS were maintained the same (15.0°, 22.6° and 29.2°). However, the NbSe_2_ PNS/CF after 20,000 cycles still exhibited an ultra-high electrochemical activity, which illustrates that the NbSe_2_ PNS/CF electrodes have a great long-term stability. To further confirm the active catalytic species in the NbSe_2_ PNS/CF electrode, the LSV curves of Nb_2_O_5_, NbSe_2_ PNS/CF, NbSe_2_ PNS/CF after 50 consecutive cyclic voltammetry sweeps and NbSe_2_ PNS/CF after 25 h stability test with the scan rate of 100 mV/s in 0.5 M H_2_SO_4_ were tested (see Figure S10). The XRD patterns of NbSe_2_ PNS/CF, NbSe_2_ PNS/CF after 50 consecutive cyclic voltammetry sweeps and NbSe_2_ PNS/CF after 25 h stability test were shown in Figure S4. Combining the XPS (Table 1) and XRD results (Figure S4), it can be concluded that the NbSe_2_ PNS in the electrode surface was gradually oxidized to Nb_2_O_5_ during the whole electrochemical test. The pure Nb_2_O_5_ exhibits a weak HER performance, which illustrates that the Nb_2_O_5_ is not an active catalytic species for hydrogen evolution reaction. The LSV curve of NbSe_2_ PNS/CF after 50 cycles almost coincided with the initial NbSe_2_ PNS/CF, while the NbSe_2_ PNS/CF after long-term stability test showed little current attenuation compared with the initial NbSe_2_ PNS/CF. The NbSe_2_ PNS/CF electrode exhibited the satisfied stability even when the electrode surface was gradually transferred to niobium oxide. Considering the low electrocatalytic performance of Nb_2_O_5_, the invariant electrocatalytic performance of NbSe_2_ PNS/CF electrode may be due to the high active of exposed NbSe_2_ catalyst.

Catalysis process is related to the interactions between the catalyst surface and the adsorbed species (reaction intermediates) [41]. Electrochemical active surface is also an important factor to reflect the electrocatalytic performance. The electrochemical double-layer capacitance (C_dl_) is used to estimate the electrochemical active surface area for each system [42]. To measure the electrochemical capacitance of CF, NbSe_2_ bulk/CF, NbSe_2_ NSs/CF and NbSe_2_ PNS/CF, CVs with a potential range of ±100 mV versus open circuit potential (OCP), were scanned at 10, 40, 80, 200 and 400 mV s^−1^ (Figure S6). The OCP for CF, NbSe_2_ NSs/CF and NbSe_2_ PNS/CF are 0.15 V, 0.21 V, 0.18 V, respectively. Figure 7e shows the slope of the current density versus the scan rate. The measured C_dl_ were plotted as a function of scan rate via a linear fitting. The C_dl_ of NbSe_2_ PNS/CF is more than twice that of NbSe_2_ NSs/CF (5.35 versus 2.3 mF cm^−2^), whereas the C_dl_ of the NbSe_2_ bulk/CF and pure CF is only 0.61 and 0.19 mF cm^−2^, respectively. These results show that NbSe_2_ PNS/CF possesses more HER active sites than that of the NbSe_2_ NSs/CF because more basal planes were exposed in this typical porous structure. Thus, this beneficial distinct feature leads to a higher HER activity. Electrochemical impedance spectroscopy (EIS) analysis was carried out to investigate the charge-transfer resistance (R_ct_) of different samples. Figure 7f shows Nyquist plots of NbSe_2_ PNS, NbSe_2_ NSs and CF. The EIS profile can be fitted to two semicircles. The first high-frequency arches are related to the solid-solid interface resistance (R_ct1_), the second semicircles in the lower frequency range are associated with the electron transfer at the solid/electrolyte interface (R_ct2_) (inset in Figure 7f) [43]. The R_S_ values of NbSe_2_ PNS/CF, NbSe_2_ NSs/CF, NbSe_2_ Bulk/CF and CF are similar (12~13 Ω), and the R_ct1_ value in each electrode is not significantly different (R_ct1_ is 7 Ω for NbSe_2_ NSs/CF, NbSe_2_ Bulk/CF and CF; R_ct1_ is 10 Ω for NbSe_2_ PNS/CF). NbSe_2_ PNS possesses a small R_ct2_ of 6.3 Ω, which is significantly lower than that of NbSe_2_ NSs of 191.7 Ω, NbSe_2_ bulk of 253.7 Ω, and CF of 298.4 Ω. The lower R_ct_ indicates the rapid HER reaction kinetics, which may be attributed to the great conductivity and abundant active edge sites of the NbSe_2_ PNS.

## 4. Discussion and Conclusions

Single-layer porous NbSe_2_ nanosheets have been prepared via double-sonication liquid-phase exfoliation with the assistance of H_2_O_2_. The single-layer porous NbSe_2_ nanosheets were loaded on the CF surface as efficient electrocatalytic electrodes for HER. Compared with the NbSe_2_ NSs/CF and pure carbon foam, the NbSe_2_ PNS/CF exhibited excellent HER catalytic properties in acidic electrolyte with a low overpotential (−50 mA cm^−2^ at an overpotential of ~148 mV), and a small Tafel slope of 75.8 mV dec^−1^. The NbSe_2_ PNS/CF shows little deactivation in continuous CV testing up to 20,000 cycles. These results suggest the promise of this novel NbSe_2_ PNS/CF electrode in electrochemical water splitting for hydrogen production. The enhanced HER performance is attributed to the accelerated electrochemical reaction that results from the increased edge active sites. The good HER performance of the NbSe_2_ PNS/CF is attributed to the increased conductivity and the faster electron-transfer rate. This work provides a new insight into the future construction of high-performance HER electrocatalysts.

## Figures and Tables

**Table 1:** The related XPS analysis results after standardization.

	NbSe_2_ NSs	NbSe_2_ PNS	NbSe_2_ PNS after 50 cycles	NbSe_2_ PNS after 25 h
Nb 3d	0.285	0.149	0.178	0.126
Se 3d	0.644	0.374	0.339	0.174
O 1s	0.071	0.476	0.482	0.696

## Supplementary Materials

The following are available online at https://www.mdpi.com/2079-4991/9/5/751/s1, Figure S1: SEM images of the carbon foam, NbSe_2_ pristine powder, NbSe_2_ NSs and NbSe_2_ PNS.; Figure S2: Typical AFM image and corresponding thickness analysis of NbSe_2_ NSs, TEM and HRTEM images of NbSe_2_ NSs; Figure S3: The high-resolution XPS spectra of NbSe_2_ NSs Se 3d and Nb 3d; Figure S4: XRD patterns of NbSe_2_ PNS, NbSe2 PNS after 50 consecutive cycle voltammetry sweeps and NbSe_2_ after 25 h stability test PNS; Figure S5: The SEM image of NbSe_2_ PNS after stability test and the TEM image of NbSe_2_ PNS after stability test; Figure S6: Double-layer capacitance measurements for determining the electrochemically active surface areas of the CF, NbSe_2_ NSs/CF and NbSe_2_ PNS/CF; Figure S7: High-resolution XPS spectrum of O 1s of NbSe_2_ PNS; Figure S8: High-resolution XPS spectrum of NbSe_2_ PNS after 50 consecutive cyclic voltammetry sweeps; Figure S9: XRD patterns of Nb_2_O_5_; Figure S10: LSV curves of Nb_2_O_5_, NbSe_2_ PNS/CF, NbSe_2_ PNS/CF after 50 consecutive cyclic voltammetry sweeps and NbSe_2_ PNS/CF after 25 h stability test. Table S1: Comparison of HER performance in acid medium for NbSe_2_ PNS/CF with other recently reported non-noble-metal related HER catalysts.
