# Supplementary material for: Active Pore-Edge Engineering of Single-Layer Niobium Diselenide Porous Nanosheets Electrode for Hydrogen Evolution
Source: Nanomaterials (Basel). 2019 May 16;9(5):751. doi: 10.3390/nano9050751 (PMC6567302; doi:10.3390/nano9050751)
Supplement: Supplementary file 1 [file nanomaterials-09-00751-s001.pdf]

Supporting Information

Active Pore-edge Engineering of Single-layer Niobium Diselenide Porous Nanosheets Electrode for Hydrogen Evolution

Jianxing Wang, Xinyue Liu, Ying Liu and Guowei Yang *

State Key Laboratory of Optoelectronic Materials and Technologies, Nanotechnology Research Center, School of Materials Science & Engineering, School of Physics, Sun Yat-sen University, Guangzhou 510275, Guangdong, China

* Corresponding author: stsygw@mail.sysu.edu.cn

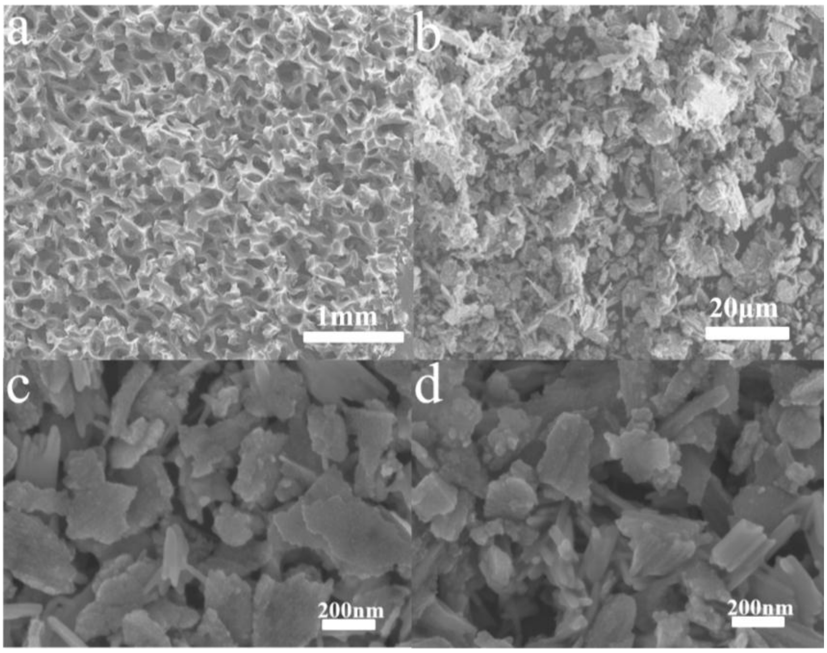

Figure S1. SEM images of (**a**) carbon foam; (**b**) $NbSe_2$ pristine powder; (**c**) $NbSe_2$ NSs; (**d**) $NbSe_2$ PNS.

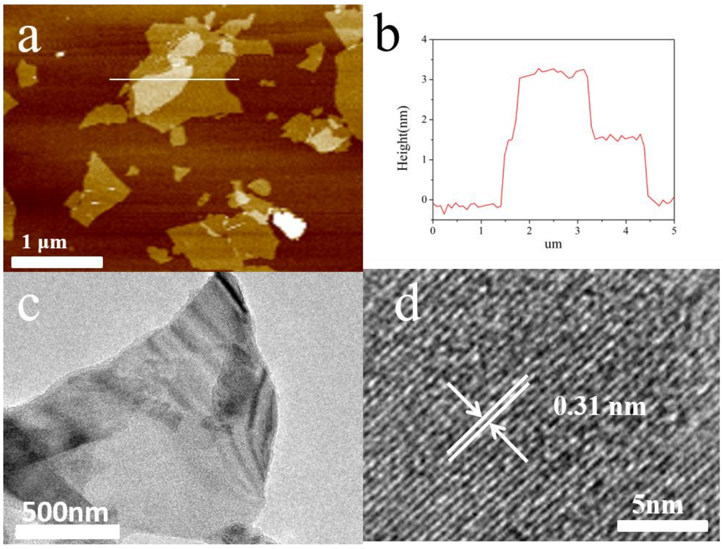

Figure S2. (**a**) Typical AFM image and (**b**) corresponding thickness analysis of $NbSe_2$ NSs. (**c**) TEM and (**d**) HRTEM images of $NbSe_2$ NSs.

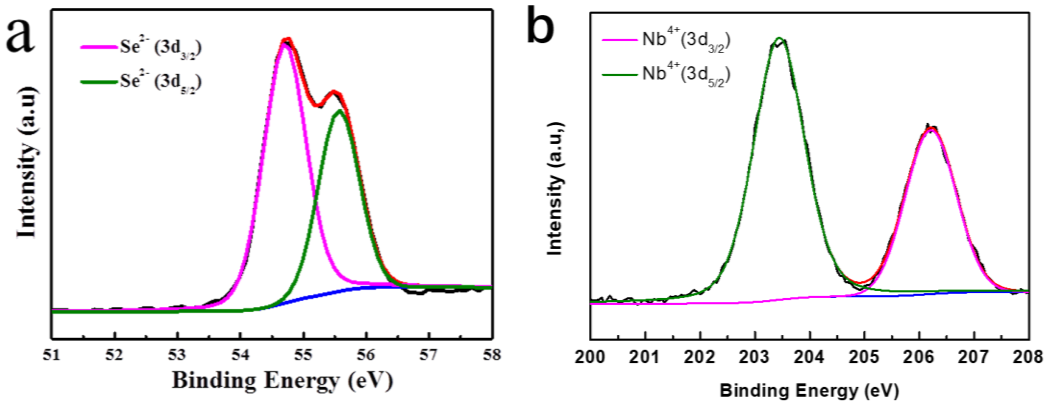

Figure S3. The high-resolution XPS spectra of $NbSe_2$ NSs (**a**) Se 3d and (**b**) Nb 3d.

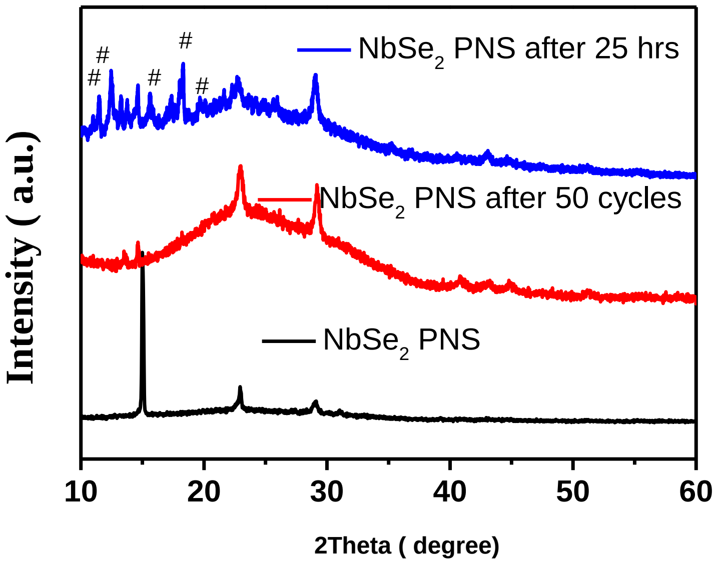

Figure S4. XRD patterns of $NbSe_2$ PNS, $NbSe_2$ PNS after 50 consecutive cycle voltammetry sweeps and $NbSe_2$ after 25 hours stability test PNS. The well number was assigned to Nb_2O_5.

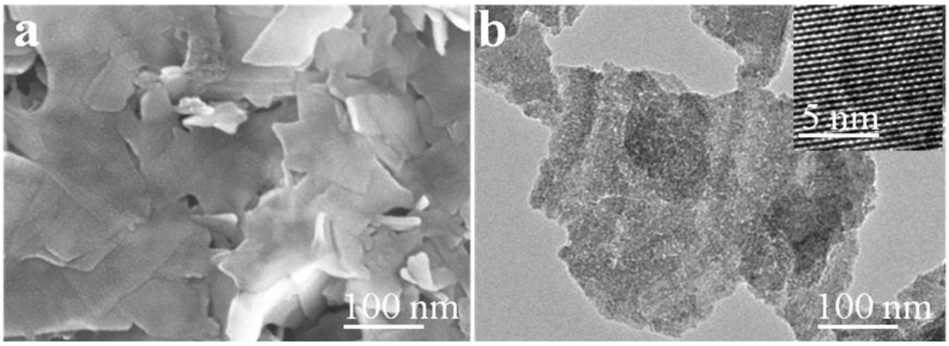

Figure S5. (**a**) The SEM image of $NbSe_2$ PNS after stability test; (**b**) the TEM image of $NbSe_2$ PNS after stability test, inset is the HRTEM image of $NbSe_2$ PNS after stability test.

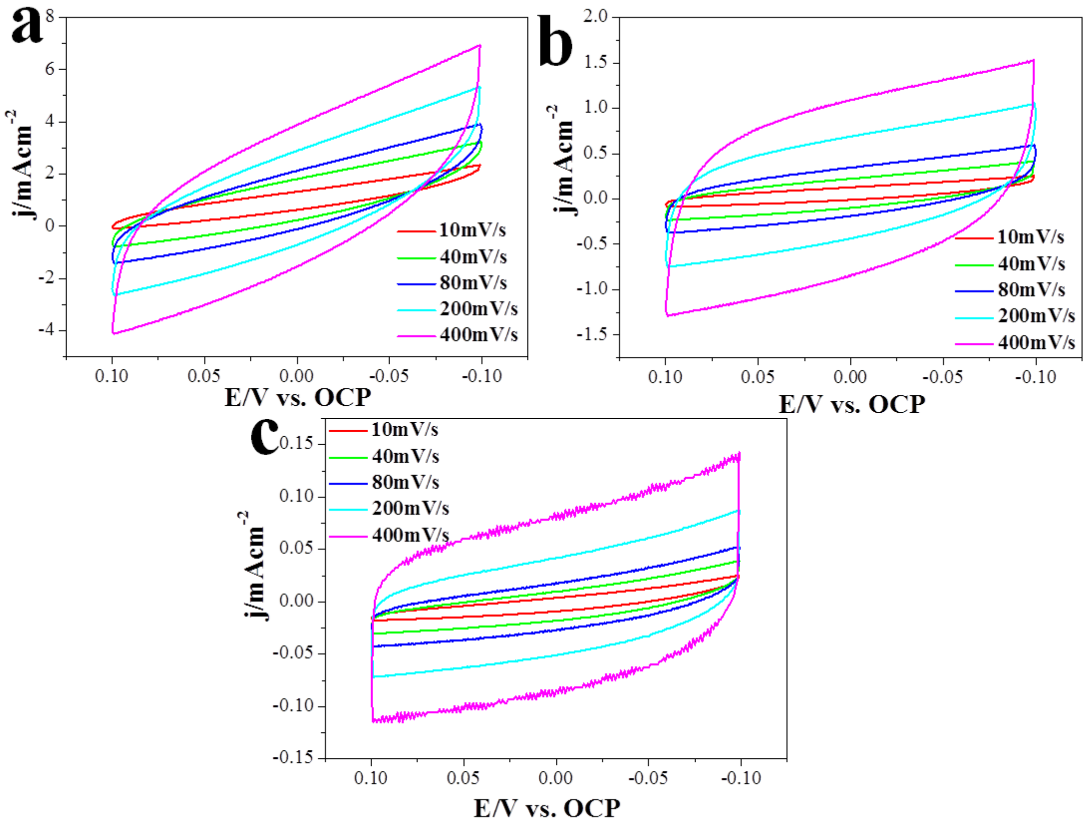

Figure S6. Double-layer capacitance measurements for determining the electrochemically active surface areas of the CF, $NbSe_2$ NSs/CF and $NbSe_2$ PNS/CF. CV curves performed across ±100 mV of the open-circuit potential (OCP) at scan rates of 10, 40, 80, 200 and 400 mV s^{-1} for the (**a**) $NbSe_2$ PNS/CF, (**b**) $NbSe_2$ NSs/CF and (**c**) CF, respectively.

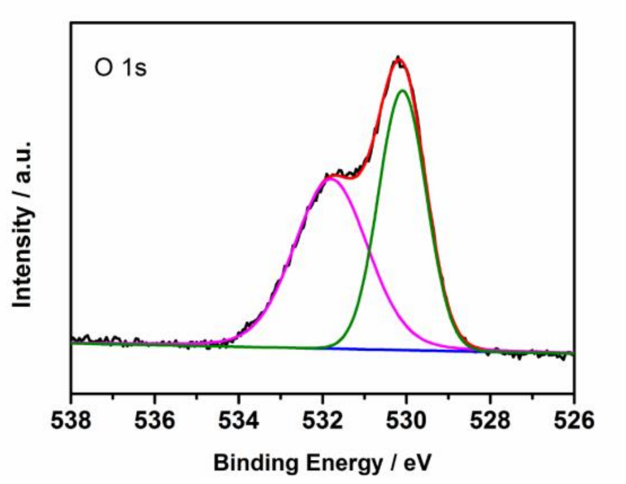

Figure S7. High-resolution XPS spectrum of O 1s of $NbSe_2$ PNS.

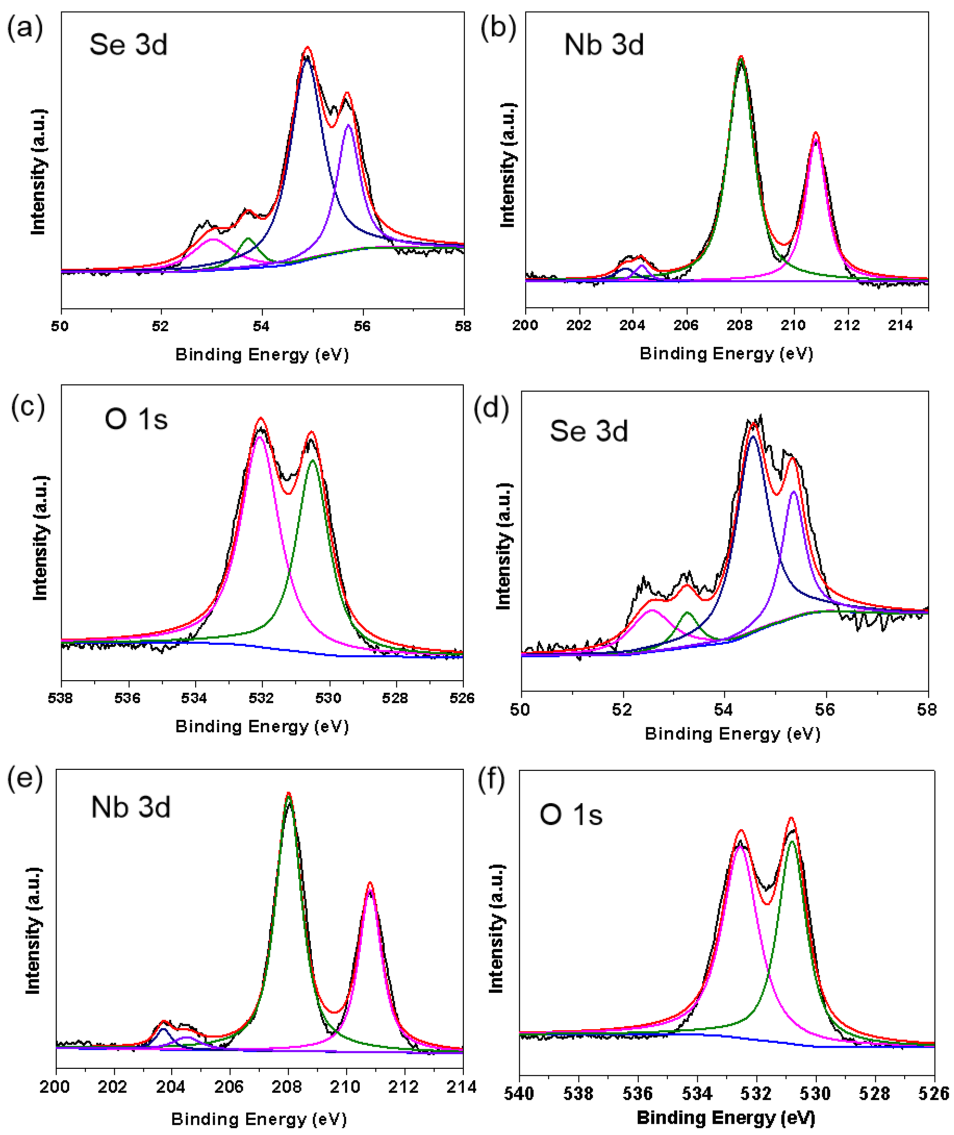

Figure S8. High-resolution XPS spectrum of $NbSe_2$ PNS after 50 consecutive cyclic voltammetry sweeps. (**a**) Se 3d, (**b**) Nb 3d and (**c**) O1s; High-resolution XPS spectrum of $NbSe_2$ PNS after 25 h stability test. (**d**) Se 3d, (**e**) Nb 3d and (**f**) O1s.

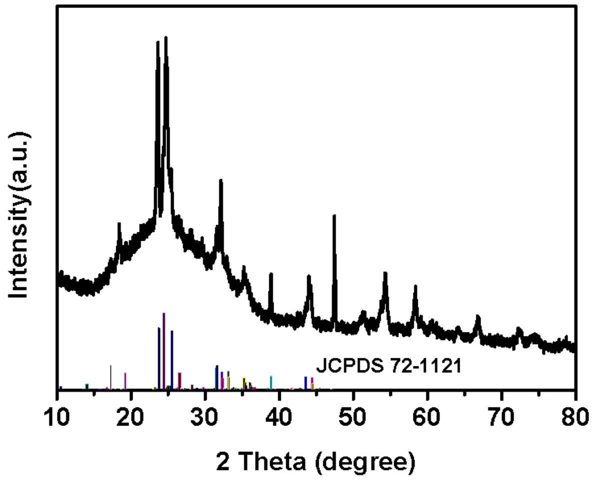

Figure S9. XRD patterns of Nb_2O_5 (JCPDS 72-1121).

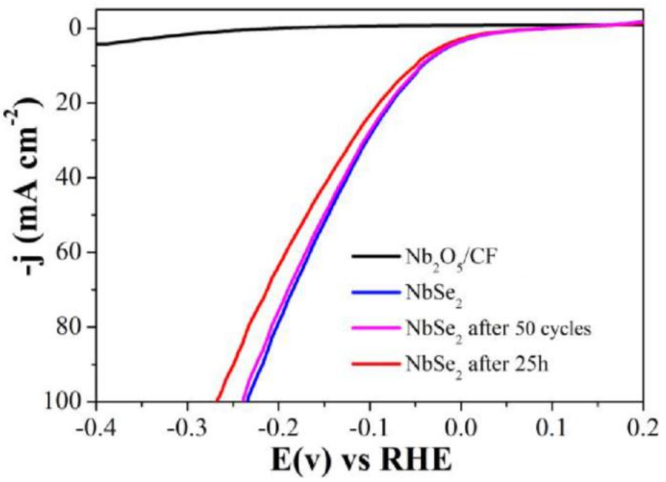

Figure S10. LSV curves of Nb_2O_5, $NbSe_2$ PNS/CF, $NbSe_2$ PNS/CF after 50 consecutive cyclic voltammetry sweeps and $NbSe_2$ PNS/CF after 25 h stability test with the scan rate of 100 mV s^{-1} in 0.5 M H_2SO_4.

Table S1. Comparison of HER performance in acid medium for $NbSe_2$ PNS/CF with other recently reported non-noble-metal related HER catalysts.

Catalyst	Tafel slope [mV dec^{-1}]	Current Density (j, mA cm^{-2})	η at the corresponding j (mV)	References
$NbSe_2$ PNS/CF	75.8	10<br>50	22<br>148	This work
$NbSe_2$	133	10	850	J. Mater. Chem. A, 4 [2016] 12241–14253
$NbSe_2$ Hybrid Nanobelts	101.2	10<br>50	450<br>520	Journal of The Electrochemical Society, 163 [2016] 384–387
MoP/CF	67.4	10<br>20	205<br>240	Appl. Catal. B 164 [2015] 144–150
WN NA/CC	92	10<br>20	198<br>265	Electrochimica Acta 154 [2015] 345–351
Three-dimensional CF/N-doped graphene@MoS2	53	10<br>50	172<br>209	J. Mater. Chem. A, 4 [2016] 12720–12725
Three-Dimensional MoS_2/GO framework	86.3	10<br>50	220<br>380	ACS Appl. Mater. Interfaces 6 [2014] 21534–21540
Self-supported porous Ni-Fe-P composite	64.6	10<br>50	89<br>145	Electrochimica Acta 219 [2016] 194–203
MoS_2 Nanosheets	43	10	187	J. Am. Chem. Soc. 135 [2013] 10274–10277
Three-Dimensional Molybdenum Sulfide Sponges	185	-	-	Small 10 [2014] 895–900
